# Dose predictions for [^177^Lu]Lu-DOTA-panitumumab F(ab′)_2_ in NRG mice with HNSCC patient-derived tumour xenografts based on [^64^Cu]Cu-DOTA-panitumumab F(ab′)_2_ – implications for a PET theranostic strategy

**DOI:** 10.1186/s41181-021-00140-1

**Published:** 2021-08-12

**Authors:** Anthony Ku, Misaki Kondo, Zhongli Cai, Jalna Meens, Min Rong Li, Laurie Ailles, Raymond M. Reilly

**Affiliations:** 1 Department of Pharmaceutical Sciences, University of Toronto, Toronto, ON Canada; 2 Princess Margaret Cancer Centre, Toronto, ON Canada; 3 Department of Medical Biophysics, University of Toronto, Toronto, ON Canada; 4 Department of Medical Imaging, University of Toronto, Toronto, ON Canada; 5 Joint Department of Medical Imaging, University Health Network, Toronto, ON Canada

**Keywords:** Panitumumab, ^64^Cu, ^177^Lu, Dosimetry, Theranostics, Head and neck squamous cell carcinoma (HNSCC)

## Abstract

**Background:**

Epidermal growth factor receptors (EGFR) are overexpressed on many head and neck squamous cell carcinoma (HNSCC). Radioimmunotherapy (RIT) with F(ab')_2_ of the anti-EGFR monoclonal antibody panitumumab labeled with the β-particle emitter, ^177^Lu may be a promising treatment for HNSCC. Our aim was to assess the feasibility of a theranostic strategy that combines positron emission tomography (PET) with [^64^Cu]Cu-DOTA-panitumumab F(ab')_2_ to image HNSCC and predict the radiation equivalent doses to the tumour and normal organs from RIT with [^177^Lu]Lu-DOTA-panitumumab F(ab')_2_.

**Results:**

Panitumumab F(ab')_2_ were conjugated to DOTA and complexed to ^64^Cu or ^177^Lu in high radiochemical purity (95.6 ± 2.1% and 96.7 ± 3.5%, respectively) and exhibited high affinity EGFR binding (K_d_ = 2.9 ± 0.7 × 10^− 9^ mol/L). Biodistribution (BOD) studies at 6, 24 or 48 h post-injection (p.i.) of [^64^Cu]Cu-DOTA-panitumumab F(ab')_2_ (5.5–14.0 MBq; 50 μg) or [^177^Lu]Lu-DOTA-panitumumab F(ab')_2_ (6.5 MBq; 50 μg) in NRG mice with s.c. HNSCC patient-derived xenografts (PDX) overall showed no significant differences in tumour uptake but modest differences in normal organ uptake were noted at certain time points. Tumours were imaged by microPET/CT with [^64^Cu]Cu-DOTA-panitumumab F(ab')_2_ or microSPECT/CT with [^177^Lu]Lu-DOTA-panitumumab F(ab')_2_ but not with irrelevant [^177^Lu]Lu-DOTA-trastuzumab F(ab')_2_. Tumour uptake at 24 h p.i. of [^64^Cu]Cu-DOTA-panitumumab F(ab')_2_ [14.9 ± 1.1% injected dose/gram (%ID/g) and [^177^Lu]Lu-DOTA-panitumumab F(ab')_2_ (18.0 ± 0.4%ID/g) were significantly higher (*P* < 0.05) than [^177^Lu]Lu-DOTA-trastuzumab F(ab')_2_ (2.6 ± 0.5%ID/g), demonstrating EGFR-mediated tumour uptake. There were no significant differences in the radiation equivalent doses in the tumour and most normal organs estimated for [^177^Lu]Lu-DOTA-panitumumab F(ab')_2_ based on the BOD of [^64^Cu]Cu-DOTA-panitumumab F(ab')_2_ compared to those estimated directly from the BOD of [^177^Lu]Lu-DOTA-panitumumab F(ab')_2_ except for the liver and whole body which were modestly underestimated by [^64^Cu]Cu-DOTA-panitumumab F(ab')_2_. Region-of-interest (ROI) analysis of microPET/CT images provided dose estimates for the tumour and liver that were not significantly different for the two radioimmunoconjugates. Human doses from administration of [^177^Lu]Lu-DOTA-panitumumab F(ab')_2_ predicted that a 2 cm diameter HNSCC tumour in a patient would receive 1.1–1.5 mSv/MBq and the whole body dose would be 0.15–0.22 mSv/MBq.

**Conclusion:**

A PET theranostic strategy combining [^64^Cu]Cu-DOTA-panitumumab F(ab')_2_ to image HNSCC tumours and predict the equivalent radiation doses in the tumour and normal organs from RIT with [^177^Lu]Lu-DOTA-panitumumab F(ab')_2_ is feasible. RIT with [^177^Lu]Lu-DOTA-panitumumab F(ab')_2_ may be a promising approach to treatment of HNSCC due to frequent overexpression of EGFR.

**Supplementary Information:**

The online version contains supplementary material available at 10.1186/s41181-021-00140-1.

## Background

Head and neck squamous cell carcinoma (HNSCC) is the 7th most prevalent cancer in the world (Bray, 2018). In the United States, HNSCC is responsible for 3% of all cancers and 1.8% of all deaths due to cancer (Siegel, 2020). In Canada, cancers of the oral cavity, esophagus or larynx were predicted to cause 8950 new cases and 4200 deaths from cancer in 2020 (Brenner, 2020). HNSCC is treated by surgery followed by radiation (50–70 Gy) or chemoradiotherapy (CRT) with high dose cisplatin (100 mg/m^2^) administered every 3 weeks for 3 cycles (Chow, 2020; De Felice et al., 2018; Schüttrumpf et al., 2020). For advanced HNSCC, this is often the definitive treatment (Chow, 2020). However, nephrotoxicity is a dose-limiting toxicity of cisplatin (Hoek et al., 2016) and grade 2 or grade 3–4 nephrotoxicity occur in up to 25% and 5–8% of patients, respectively (Saba et al., 2017). Reduction in the dose of cisplatin (< 50 mg/m^2^) or substituting carboplatin may reduce these toxicities but is associated with poorer survival.

The epidermal growth factor receptor (EGFR) is overexpressed on 38–47% of HNSCC tumours and is a poor prognostic marker (Kalyankrishna and Grandis, 2006). Combining anti-EGFR chimeric monoclonal antibody (mAb) cetuximab (Erbitux®, Eli Lilly) with platinum-based chemotherapy and 5-fluorouracil (EXTREME regimen) showed modest improvement in overall survival (Vermorken et al., 2008) and this regimen is recommended in the National Comprehensive Cancer Network Guidelines for treatment of HNSCC (Colevas, 2018). Unfortunately, patients with recurrent or metastatic HNSCC resistant to this regimen have a poor prognosis and there is no accepted second line treatment. The fully human anti-EGFR mAb panitumumab (Vectibix, Amgen) has also shown promise for improving the outcome of patients with human papillomarvirus (HPV)-negative HNSCC treated with CRT (Ferris et al., 2016).

Radioimmunotherapy (RIT) which links the β-particle-emitting radionuclide ^177^Lu [t_1/2_ = 6.7 d; Eβ_max_ = 0.5 MeV (78.6%), Eβ_max_ = 0.38 MeV (9.1%), Eβ_max_ = 0.18 MeV (12.2%)] to anti-EGFR mAbs may be a promising approach for HNSCC, considering that EGFR are frequently overexpressed on tumours (Kalyankrishna and Grandis, 2006). RIT may also prove more effective than “naked” anti-EGFR mAbs, since it does not rely on inhibiting tumour growth signaling, but rather on inflicting lethal DNA damage. The feasibility of RIT for HNSCC is suggested by preclinical studies of cetuximab or panitumumab modified with DOTA (1,4,7,10-tetraazacyclododecane-1,4,7,10-tetraacetic acid) to complex [^177^Lu] Lu, which reported strong tumour growth inhibition of HNSCC xenografts in BALB/c nude mice or athymic mice that were resistant to cetuximab or panitumumab (Liu et al., 2014; Song et al., 2017). We propose that patients with HNSCC could be selected for RIT with [^177^Lu]Lu-labeled anti-EGFR mAbs by positron emission tomography (PET) with the corresponding [^64^Cu]Cu-labeled mAbs [Eβ^+max^ = 0.65 MeV (17.4%)] in a “PET theranostic” strategy. An analogous SPECT theranostic strategy that uses [^177^Lu]Lu-labeled panitumumab to select patients for RIT is not as attractive due to the low abundance γ-photons emitted by ^177^Lu [Eγ = 113 keV (6%) and 208 keV (10%)] which combined with the collimation required for SPECT decreases the sensitivity for tumour imaging. In addition, the higher absorbed doses for ^177^Lu compared to ^64^Cu are not suitable for a theranostic approach in which imaging is performed to select patients for RIT. Although many HNSCC are EGFR-positive, PET with [^64^Cu]Cu-labeled mAbs would confirm lesions as EGFR-positive and importantly predict the tumour and normal tissue uptake of [^177^Lu]Lu-labeled anti-EGFR mAbs. Moreover, information on the tumour and normal organ uptake can be used to estimate the radiation equivalent doses from RIT which may predict the effectiveness and normal organ toxicity in an individual patient. Since ^64^Cu has a short half-life (t_1/2_ = 12.7 h), F(ab')_2_ fragments of mAbs that accumulate in tumours but are quickly eliminated from the blood and most normal organs in the feasible imaging time of ^64^Cu [up to 24 h post-injection (p.i.)] would be most attractive for a PET theranostic strategy. F(ab')_2_ also offer advantages for RIT because their more rapid elimination compared to IgG quickly decreases circulating radioactivity which is responsible for dose-limiting bone marrow toxicity from RIT due to the cross-fire effect from the 2 mm β-particles emitted by ^177^Lu (Aghevlian et al., 2017). Moreover, F(ab')_2_ unlike Fab do not exhibit high uptake in the kidneys, which minimizes kidney toxicity from RIT (Behr et al., 1996). We previously reported that panitumumab F(ab')_2_ complexed to ^64^Cu by NOTA (2,2′,2″-(1,4,7-triazacyclononane-1,4,7-triyl) triacetic acid) imaged s.c. PANC-1 human pancreatic cancer tumours that overexpress EGFR in non-obese diabetic severe combined immunodeficiency (NOD/scid) mice (Boyle et al., 2015). This study further showed that radioactivity in the blood in non-tumour bearing Balb/c mice was much lower at 18 h p.i. of [^64^Cu]Cu-NOTA-panitumumab F(ab')_2_ and Fab than for the intact IgG, but kidney uptake was much higher for Fab than F(ab')_2_, thus F(ab')_2_ are preferred for PET and RIT. [^177^Lu]Lu-DOTA-cetuximab F(ab')_2_ have been reported to be effective for RIT of human colorectal xenografts in female SWISS nu/nu mice (Bellaye et al., 2018).

For a PET theranostic approach to be feasible, the tumour and normal organ uptake of [^64^Cu]Cu-DOTA-panitumumab F(ab')_2_ must be very similar to that of [^177^Lu]Lu-DOTA-panitumumab F(ab')_2_. Moreover, the radiation equivalent doses in the tumour and normal organs predicted for [^177^Lu]Lu-DOTA-paniumumab F(ab')_2_ based on the biodistribution (BOD) of [^64^Cu]Cu-DOTA-panitumumab F(ab')_2_ assessed by PET need to accurately predict those estimated directly from the BOD of [^177^Lu]Lu-DOTA-panitumumab F(ab')_2_. Our aim was to compare the BOD of [^64^Cu]Cu-DOTA-panitumumab F(ab')_2_ and [^177^Lu]Lu-DOTA-panitumumab F(ab')_2_ in NOD-*Rag1*^*null*^ *IL2rg*^*null*^ (NRG) mice with subcutaneous (s.c.) patient-derived HNSCC tumour xenografts (PDX) and estimate the radiation equivalent doses in the tumour and normal organs for [^177^Lu]Lu-DOTA-panitumumab F(ab')_2_ based on the tumour and normal organ uptake of [^64^Cu]Cu-DOTA-panitumumab F(ab')_2_ assessed in BOD studies or by region-of-interest (ROI) analysis of microPET/CT images. We compared these predicted doses based on [^64^Cu]Cu-DOTA-panitumumab F(ab')_2_ to those estimated directly from the BOD of [^177^Lu]Lu-DOTA-panitumumab F(ab')_2_ to assess the feasibility of a PET theranostic strategy. The PDX mouse model of HNSCC used in this study is clinically relevant because these PDX recapitulate the properties of HNSCC tumours in patients (Karamboulas and Ailles, 2019). This strengthens our assessment of the feasibility of a PET theranostic strategy for patients with HNSCC.

## Methods

### Cell culture and patient-derived HNSCC xenografts

MDA-MB-468 human breast cancer cells (1.3 × 10^6^ EGFR/cell) (Reilly and Gariepy, 1998) were purchased from the American Type Culture Collection (ATCC, Manassas, VA) and cultured in RPMI 1640 medium (Sigma-Aldrich, St. Louis, MO) supplemented with 10% fetal bovine serum (FBS; Invitrogen, Carlsbad, CA) and 1% penicillin streptomycin (Sigma-Aldrich). A primary tumour specimen (#391) was surgically obtained from a patient with HNSCC under a protocol approved by the Research Ethics Board at the University Health Network (Protocol No. 12–5639). This tumour was dissected into small fragments (~ 1 mm^3^) and engrafted subcutaneously (s.c.) on the right flank of NOD-*Rag1*^*null*^ *IL2rg*^*null*^ (NRG) mice. These patient-derived tumour xenografts (PDX) were serially propagated in NRG mice following an Animal Care Protocol (No. 1542.28) approved by the Animal Care Committee at the University Health Network and following Canadian Council on Animal Care guidelines. The PDX used in this study were between the 3rd to 5th passage from the initial engraftment of the HNSCC tumour in NRG mice.

### Panitumumab and trastuzumab F(ab´)_2_

Panitumumab F(ab´)_2_ (MW ~ 110 kDa) were produced by proteolytic digestion of panitumumab IgG (Vectibix®, Amgen, Thousand Oaks, CA) using immobilized pepsin (Pierce Biotechnology, Rockford, IL) as reported with minor modifications (Boyle et al., 2015). Briefly, panitumumab IgG was buffer-exchanged into 20 mM sodium acetate buffer (pH 4.5) by ultrafiltration on an Amicon ultracentrifugal unit (Millipore, Burlington, MA; MWCO = 30 kDa). Panitumumab IgG was then incubated at a ratio of 4 mg of IgG per 0.25 mg of immobilized pepsin resin in 0.25 mL slurry at 37 °C for 5 h in a Excella E24 Incubator Shaker (New Brunswick Scientific, Edison, NJ) at 300 rpm. Following digestion, the resin suspension was rinsed with ice cold phosphate buffered saline (PBS), pH 7.4, and centrifuged at 1000×g for 5 min and the supernatant collected. This was repeated 2 times and the pooled supernatants were filtered through a Millex®-GV PDVF 0.22 μm filter to remove residual resin. F(ab´)_2_ were re-concentrated to 20.0–24.5 mg/mL and buffer-exchanged into 100 mM NaHCO_3_ buffer, pH 8.2 on an Amicon ultracentrifugal unit (MWCO = 30 kDa). The 100 mM NaHCO_3_ buffer, pH 8.2 buffer was purified from trace metals by passage through a column of Chelex-100 cation exchange resin (BioRad, Mississauga, ON, Canada). The F(ab´)_2_ concentration was measured spectrophotometrically at 280 nm (A_280_ of a 1 mg/mL solution = 1.40). The purity and homogeneity of F(ab´)_2_ were assessed by sodium dodecyl sulfate polyacrylamide gel electrophoresis (SDS-PAGE) on a 7.5% Mini-Protean Tris/glycine mini-gel (BioRad) under reducing (2-mercaptoethanol) and non-reducing conditions with bands stained with Biosafe Coomassie Blue G-250 (BioRad). Anti-human epidermal growth factor receptor-2 (HER2) trastuzumab F(ab´)_2_ was prepared by proteolytic digestion of trastuzumab IgG (Herceptin, Roche, Mississauga, ON, Canada).

### Labeling of F(ab´)_2_ with ^64^Cu or ^177^Lu

Panitumumab F(ab´)_2_ (2 mg; 20.0–24.5 mg/mL) in 100 mM NaHCO_3_ buffer, pH 8.2 were conjugated to 1,4,7,10-tetraazacyclododecane-1,4,7,10-tetraacetic acid (DOTA) by reaction with a 20-fold molar excess of the N-hydroxysuccinimide ester (DOTA-NHS; Macrocyclics, Dallas, TX) for 2 h at room temperature (RT) on a nutating mixer (VWR, Mississauga, ON, Canada). DOTA-panitumumab F(ab´)_2_ were purified from excess DOTA and buffer-exchanged into 100 mM HEPES, pH 5.5 and re-concentrated to 20–28.5 mg/mL using an Amicon ultracentrifugal unit (MWCO = 10 kDa) centrifuged at 7500×g for 5 mins. This was repeated 8 times to ensure complete removal of excess DOTA. The DOTA substitution level [moles DOTA/mole F(ab´)_2_] was determined by trace-labeling an aliquot (100 μg; 5 μL) of the impure conjugation mixture with [^64^Cu]CuCl_2_ (Washington University, St. Louis, MO; 40 MBq/μL) and measuring the proportion of [^64^Cu]Cu-DOTA-panitumumab F(ab´)_2_ and [^64^Cu]Cu-DOTA by instant thin layer-silica gel chromatography (ITLC-SG; Agilent Technologies, Santa Clara, CA) developed in 100 mM sodium citrate, pH 5.5, then multiplying this proportion by the 20:1 M ratio of NHS-DOTA:F(ab´)_2_ in the reaction (Reilly, 2007). The R_f_ values of [^64^Cu]Cu-DOTA-panitumumab F(ab´)_2_ and free [^64^Cu]Cu-DOTA (or ^64^Cu) were 0.0 and 1.0, respectively.

Purified DOTA-panitumumab F(ab´)_2_ (~ 600 μg in 30 μL) were labeled with ^64^Cu to a specific activity of 0.074–1.7 MBq/μg by incubation with [^64^Cu]CuCl_2_ (40 MBq/μL; Washington University) for 1 h at 42 °C. No post-labeling purification was performed. The radiochemical purity (RCP) of [^64^Cu]Cu-DOTA-panitumumab F(ab´)_2_ was confirmed by ITLC and size exclusion (SE)-HPLC on an Agilent 1260 Infinity II HPLC system fitted with a BioSep SEC-S4000 column (300 × 7.8 mm; Phenomenex, Torrance, CA) eluted with NaH_2_PO_4_ buffer, pH 7.0 at a flow rate of 0.50 mL/min with UV detection (λ = 280 nm) and radioactivity detection by a Flowstar LB514 radioactivity detector fitted with a BGO-X flow cell (Berthold Technologies, Bad Wildbad, Germany). DOTA-panitumumab F(ab´)_2_ (~ 600 μg in 30 μL) were labeled at a SA = 0.07–0.2 MBq/μg with ^177^Lu by incubation wih [^177^Lu]LuCl_3_ (McMaster University, Hamilton, ON, Canada) at 42 °C for 3 h. The RCP of [^177^Lu]Lu-DOTA-panitumumab F(ab´)_2_ was determined by ITLC as described for [^64^Cu]Cu-DOTA-panitumumab F(ab´)_2_. Trastuzumab F(ab´)_2_ were similarly conjugated to DOTA and labeled with ^177^Lu.

### EGFR immunoreactivity

A saturation radioligand binding assay was performed to measure the dissociation constant (K_d_) and maximum number of binding sites (B_max_) for binding of [^177^Lu]Lu-DOTA-panitumumab F(ab´)_2_ to EGFR on MDA-MB-468 human breast cancer cells (1.3 × 10^6^ EGFR/cell) (Reilly and Gariepy, 1998). Increasing concentrations of [^177^Lu]Lu-DOTA-panitumumab F(ab´)_2_ (0.098–200 nmoles/L) were incubated with 1 × 10^6^ MDA-MB-468 cells in 200 μL of PBS, pH = 7.4 in 1.5 mL Eppendorf tubes at 4 °C for 3 h with gentle shaking every 30 mins. The samples were then centrifuged at 1000×g for 2 mins on an Eppendorf Centrifuge 5424 (Thermo Fisher Scientific, Waltham, MA) and the supernatant containing unbound ^177^Lu collected. The cell pellets were rinsed with ice cold PBS, pH 7.4, centrifuged again and the supernatant was collected and pooled with the previously collected supernatant. This was repeated twice. Total bound ^177^Lu (TB) in the cell pellets and unbound ^177^Lu in the supernatant were measured in a γ-counter (Model 1480; PerkinElmer, Waltham, MA). Non-specifically bound ^177^Lu (NSB) was assessed by repeating the assay in the presence of 50-fold molar excess of panitumumab IgG. Specifically bound ^177^Lu (SB) was calculated by subtracting NSB from TB. [^177^Lu]Lu-DOTA-panitumumab F(ab´)_2_ bound to MDA-MB-468 cells (pmoles) was plotted vs. the concentration of free (unbound) [^177^Lu]Lu-DOTA-panitumumab F(ab´)_2_ (nmoles/L) and the curve was fitted to a one-site-receptor-binding model using Prism Ver. 4.0 software (GraphPad, San Diego, CA).

### Biodistribution (BOD) studies and microPET/CT

The tumour and normal tissue uptake of [^64^Cu]Cu-DOTA-panitumumab F(ab´)_2_ were determined by BOD and microPET/CT studies in NRG mice with subcutaneous (s.c.) HNSCC PDX. Groups of 3–4 tumour-bearing NRG mice were injected i.v. (tail vein) with [^64^Cu]Cu-DOTA-panitumumab F(ab´)_2_ (5.5–14.0 MBq; 50 μg) and sacrificed at 6 h, 24 or 48 h post-injection (p.i.). The tumour and samples of blood and normal tissues were obtained, weighed and the radioactivity in each measured in a γ-counter using a window (425–640 keV) to include the 511 keV annihilation γ-photon of ^64^Cu. Tumour and normal organ uptake were expressed as percent injected dose/g (%ID/g). MicroPET/CT was performed after i.v. injection (tail vein) of 37 MBq (50 μg) of [^64^Cu]Cu-DOTA-panitumumab F(ab´)_2_ in a group of 4 NRG mice with s.c. HNSCC PDX. Mice were anaesthetized using 2% isoflurane in O_2_ and were imaged in a supine position on a NanoScan® SPECT/CT/PET system (Mediso, Budapest, Hungary). PET images were acquired for 10, 20 and 40 mins at 6, 24 and 48 h p.i. of [^64^Cu]Cu-DOTA-panitumumab F(ab´)_2_, respectively. Images were reconstructed by an ordered subset expectation maximization (OSEM) algorithm and consisted of 4 subsets and 4 iterations with attenuation and scatter correction supported by an isotropic voxel size of 300 μm. Prior to PET, CT images were acquired with 50 kVp X-rays, 980 μA and 300 msec exposure time. CT scans were reconstructed using the medium voxel and slice thickness settings, resulting in an isotropic voxel size of 250 μm. PET and CT images were co-registered by the Mediso Nucline NanoScan 3.00.020.0000 software. PET and CT DICOM files were exported using Mediso’s Nucline Acquisition/Reconstruction Software to the Inveon Research Workplace Software 4.0 (Siemens) for analysis and quantification of ^64^Cu uptake (%ID/g) as well as estimation of the volume of the liver and tumour. Regions of interest (ROI) were drawn around the tumour and liver on the PET images aided by delineation of the anatomy on the CT images to compare the accuracy of PET for quantifying the tumour and liver uptake compared to BOD studies. The ROI for each organ was drawn two-dimensionally on axial slices of the image. To ensure the coverage of the entire organ, > 5 ROIs were drawn before the slices were convoluted to form a 3D volume that encompassed the entire organ.

### Biodistribution (BOD) studies and microSPECT/CT

The tumour and normal tissue uptake of [^177^Lu]Lu-DOTA-panitumumab F(ab´)_2_ was determined by BOD and microSPECT/CT studies. [^177^Lu]Lu-DOTA-panitumumab F(ab´)_2_ (6.5 MBq; 50 μg) were injected i.v. (tail vein) in NRG mice with s.c. HNSCC PDX and groups (*n* = 3–4) of mice were sacrificed at 6 h, 24 h or 48 h p.i. The tumour, and samples of blood and other tnormal issues were obtained, weighed and counted in a γ-counter using a window (130–470 kev) to include the γ-photons of ^177^Lu [Eγ = 113 keV (6.6%) and Eγ = 208 keV (11%)]. Tumour and normal organ uptake was expressed as %ID/g. The excised tumours were subjected to immunohistochemical (IHC) staining with anti-human EGFR antibodies (Invitrogen, Carlsbad, CA; Cat. No. 28–8763) to confirm EGFR positivity. In addition, the tumour and normal tissue BOD of irrelevant [^177^Lu]Lu-DOTA-trastuzumab F(ab´)_2_ (2.90 MBq; 50 μg) were determined in a separate group of 5 NRG mice bearing HNSCC PDX at 24 h p.i. The excised tumours in these mice were stained for HER2 using anti-human HER2 antibodies (Invitrogen Cat. No. MA5–14509). Representative mice were anaesthetized using 2% isoflurane in O_2_ and microSPECT/CT images were acquired in a supine position, at 6 h, 24 h and 48 h p.i. of [^177^Lu]Lu-DOTA-panitumumab F(ab´)_2_ on a NanoScan® SPECT/CT/PET system (Mediso). Images were acquired in a 256 × 256 matrix. A window (± 10%) was set around each of the *γ*-photopeaks (208.4 keV; 112.9 keV; 56.1 keV) of ^177^Lu. A Mediso APT62 collimator (WB-HS standard) was affixed to each of the 4 detector NaI (TI) detector heads. Images were reconstructed by Monte Carlo methods with three subsets of data undergoing 48 iterations using the Mediso Nucline NanoScan acquisition and reconstruction software (ver 3.00.020.0000). Prior to SPECT, imaging CT images were acquired with 50 kVp X-rays, 980 μA and a 300 msec exposure time. CT scans were reconstructed using the medium voxel and slice thickness settings resulting in an isotropic voxel size of 250 μm. SPECT and CT were co-registered by the Mediso Nucline acquisition/reconstruction software. MicroSPECT/CT images were similarly obtained for NRG mice with HNSCC PDX at 24 h p.i. of [^177^Lu]Lu-DOTA-trastuzumab F(ab´)_2_. All animal studies were conducted under a protocol (AUP 2843.8) approved by the Animal Care Committee at the University Health Network following Canadian Council on Animal Care guidelines.

### Tumour and normal organ dosimetry

The radiation equivalent doses in NRG mice with s.c. HNSCC PDX after i.v. injection of [^64^Cu]Cu-DOTA-panitumumab F(ab´)_2_ or [^177^Lu]Lu-DOTA-panitumumab F(ab´)_2_ were estimated from the tumour and normal organ uptake of activity in source organs measured in BOD studies. The mean equivalent dose (D) was calculated as D = Ã_s_ × S × W_R_, where Ã_s_ is the time-integrated activity in the source organs or tumour and S are the Snyder values for mice (Bitar et al., 2007; Xie and Zaidi, 2013) and W_R_ is the radiation weighting factor. W_R_ is 1 for x-rays, γ rays and β-particles, thus taken as 1 in this study. Ã_s_ in the source organs or in the tumour were estimated using Prism Ver. 4.0 software (GraphPad) from the area-under-the-curve (AUC) up to 48 h p.i. of [^64^Cu]Cu-DOTA-panitumumab F(ab´)_2_ or [^177^Lu]Lu-DOTA-panitumumab F(ab´)_2_ (AUC_0–48 h;_ Bq × sec) derived from the activity vs. time curves. The activity/source organ at each time point t (s) was calculated using the formula %ID/g (t) × ID/100 × organ weight × exp.(−kt), where %ID/g was obtained from the BOD studies, ID was the injected dose in Bq, and k was the decay constant for ^64^Cu (1.52 × 10^− 5^ s^− 1^) or ^177^Lu (1.21 × 10^− 6^ s ^− 1^). The time integrated activity from 48 h p.i. to infinity (A_48 h – ∞_; Bq × sec) was calculated by dividing the activity at 48 h p.i. by the decay constant for ^64^Cu or ^177^Lu, assuming further elimination of activity from source organs only by radioactive decay. The S-value for the tumour was estimated using the sphere model in OLINDA/EXM software based on the measured tumour mass (Stabin et al., 2005). This was repeated with the estimated uptake in the tumour and liver determined by ROI analysis of the microPET/CT images at 6 h, 24 h and 48 h p.i. of [^64^Cu]Cu-DOTA-panitumumab F(ab´)_2_. To predict the doses in normal organs in humans, the activity in source organs of a human adult female with a 2 cm diameter spherical tumour in the neck at the different times up to 48 h p.i. of the RICs were estimated based on proportional extrapolation of the activities in mice using the % kg/g method, i.e. (%ID/organ)_human_ = [(%ID/organ)_mouse_ × (mouse body weight/human body weight)] (Kirschner et al., 1973). Mouse and human body weight used in the calculation were set to 30 g and 56,900 g, respectively. The time-integrated activity from 0 to 48 h p.i. (AUC_0–48 h;_ Bq × sec) and from 48 h p.i. to infinity (A_48 h – ∞_; Bq × sec) were calculated as described earlier for mice, and the combined A_0h to ∞_ for each human source organ was used to predict the equivalent doses in human female adults (mSv/Bq) using OLINDA/EXM 1.0 software (Stabin et al., 2005).

### Statistical analysis

All results were reported as mean ± SEM. Statistical comparisons for normal organ and tumour uptake of [^64^Cu]Cu-DOTA-panitumumab F(ab´)_2_ and [^177^Lu]Lu-DOTA-panitumumab F(ab´)_2_ were made using a Mann-Whitney U test (*P* < 0.05) and Prism 4.0 software (GraphPad). Statistical comparisons for radiation equivalent dose were made using a two-tailed unpaired Student’s t-test (*P* < 0.05) using PrismVersion 4.0 software (GraphPad).

## Results

### [^64^Cu]cu- or [^177^Lu]Lu-DOTA-panitumumab F(ab´)_2_

**Fig. 1:**
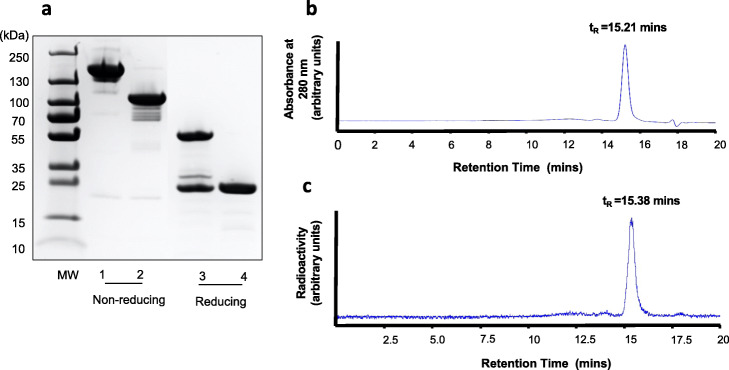
**a.** SDS-PAGE analysis of panitumumab IgG and F(ab´)_2_ under non-reducing (lanes 1, 2, respectively) or reducing (lanes 3, 4, respectively) conditions on a 7.5% Tris/Glycine mini-gel stained with Coomassie blue. **b.** SE-HPLC chromatogram of [^64^Cu]Cu-DOTA-panitumumab F(ab´)_2_ analyzed on a BioSep SEC-4000 column eluted with NaH_2_PO_4_ buffer, pH 7.0 at a flow rate of 0.5 mL/min with UV detection at 280 nm. C. SE-HPLC chromatogram of [^64^Cu]Cu-DOTA-panitumumab F(ab´)_2_ with radioactivity detection. Integration of the peak with t_R_ = 15.21 mins on the chromatogram with UV detection indicated a purity for DOTA-panitumumab F(ab´)_2_ of 96.1%. Integration of the peak with t_R_ = 15.38 mins on the chromatogram with radioactivity detection indicated a radiochemical purity for [^64^Cu]Cu-DOTA-panitumumab F(ab´)_2_ of 96.3%. There is a slight delay between detection of the peak by the radioactivity detector compared to the UV detector since these detectors are in sequence

Panitumumab F(ab´)_2_ were produced by proteolytic digestion of the IgG using immobilized pepsin. SDS-PAGE analysis (Fig. 1a) under non-reducing conditions revealed one major band for F(ab´)_2_ corresponding to the expected M_r_ ~ 110 kDa, while panitumumab IgG migrated as a band with M_r_ ~ 150 kDa. Under reducing conditions, F(ab´)_2_ migrated as one major band at M_r_ ~ 24 kDa, corresponding to the variable and the constant region of the heavy chain (V_H_-C_H_) and the light chain (V_L_-C_L_). Panitumumab IgG migrated as two major bands under reducing conditions with M_r_ = 50 kDa and 24 kDa, corresponding to the dissociated heavy and light chains, respectively.

**Fig. 2:**
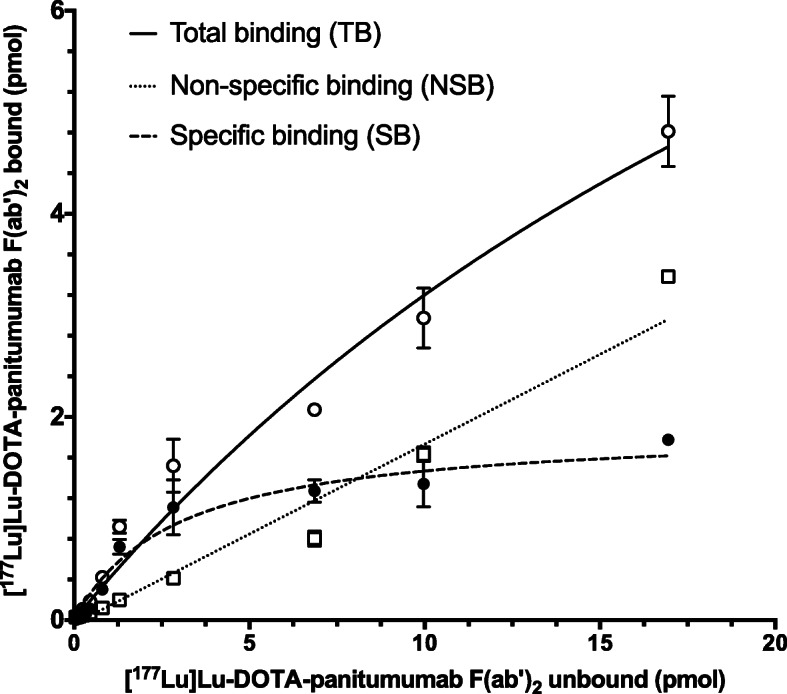
Binding of [^177^Lu]Lu-DOTA-panitumumab F(ab´)_2_ to EGFR-overexpressing MDA-MB-468 human breast cancer cells in the absence (total binding; TB) or presence (non-specific binding; NSB) of an excess (2500 nmoles/L) of panitumumab IgG. Specific binding (SB) was calculated by subtracting NSB from TB. Curves were fitted to a one-site receptor binding model using Prism Ver. 4.0 software (GraphPad). Error bars represent the mean ± SD of duplicate samples. In the representative assay shown, K_d_ = 2.9 ± 0.7 × 10^− 9^ mol/L and B_max_ = 1.1 ± 0.1 × 10^6^ EGFR/cell

SE-HPLC with UV detection at 280 nm demonstrated a single peak for panitumumab F(ab´)_2_ with a retention time (t_R_) = 15.21 mins (Fig. 1b). Panitumumab F(ab´)_2_ were conjugated to 3.8 ± 0.4 DOTA per F(ab´)_2_. DOTA-panitumumab F(ab´)_2_ were labeled with ^64^Cu or ^177^Lu to a RCP of 95.6 ± 2.1% and 96.7 ± 3.5%, respectively, measured by ITLC-SG developed in 100 mM sodium citrate, pH 5.5. SE-HPLC confirmed high RCP for these RICs with a single peak a t_R_ = 15.38 mins for [^64^Cu]Cu-DOTA-panitumumab F(ab´)_2_ (Fig. 1c) and [^177^Lu]Lu-DOTA-panitumumab F(ab´)_2_ (not shown). [^177^Lu]Lu-DOTA-panitumumab F(ab´)_2_ exhibited saturable binding to EGFR on MDA-MB-468 cells that was displaced by a 50-fold molar excess of panitumumab IgG (Fig. 2). Fitting of the SB curve to a one-site receptor binding model revealed a K_d_ = 2.9 ± 0.7 × 10^− 9^ mol/L indicating high affinity binding to EGFR. The B_max_ for MDA-MB-468 cells was 1.1 ± 0.1 × 10^6^ EGFR/cell which was similar that reported for binding of EGF or anti-EGFR mAb 528 labeled with ^111^In to these cells (1.3 × 10^6^ EGFR/cell) (Reilly et al., 2000). Trastuzumab F(ab´)_2_ was produced similarly and derivatized with 3.3 ± 0.02 DOTA which enabled labeling with ^177^Lu to high RCP (97.4 ± 0.1%) measured by ITLC-SG. SDS-PAGE analysis (Fig. S1) revealed a major protein band with M_r_ ~ 100 kDa for trastuzumab F(ab´)_2_and a major band with the expected M_r_ ~ 150 kDa for trastuzumab IgG under non-reducing conditions. Under reducing conditions, trastuzumab F(ab´)_2_ migrated as a single band at M_r_ ~ 25 kDa, corresponding to the variable and constant region of the heavy chain (V_H_-C_H_) and the light chain (V_L_-C_L_) while trastuzumab IgG migrated as two main bands with M_r_ ~ 50 kDa and 25 kDa, corresponding to the dissociated heavy and light chains, respectively.

### MicroPET/CT and microSPECT/CT

**Fig. 3:**
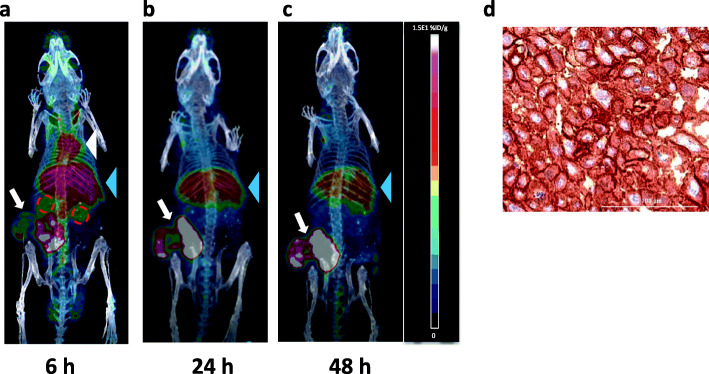
Posterior whole-body coronal microPET/CT images of a NRG mouse bearing s.c. implanted HNSCC PDX at **a.** 6 h, **b.** 24 h or **c.** 48 h p.i. of [^64^Cu]Cu-DOTA-panitumumab F(ab´)_2_. The tumour is shown by the white arrow. At 6 h p.i., the mediastinum (blood pool; white arrowhead), liver (blue arrowhead) and kidneys (broken red circles) are visualized, while at 24 and 48 h p.i., the liver was the only normal organ visualized. **d.** Immunohistochemical staining of the excised tumour demonstrated strong EGFR positivity

**Fig. 4:**
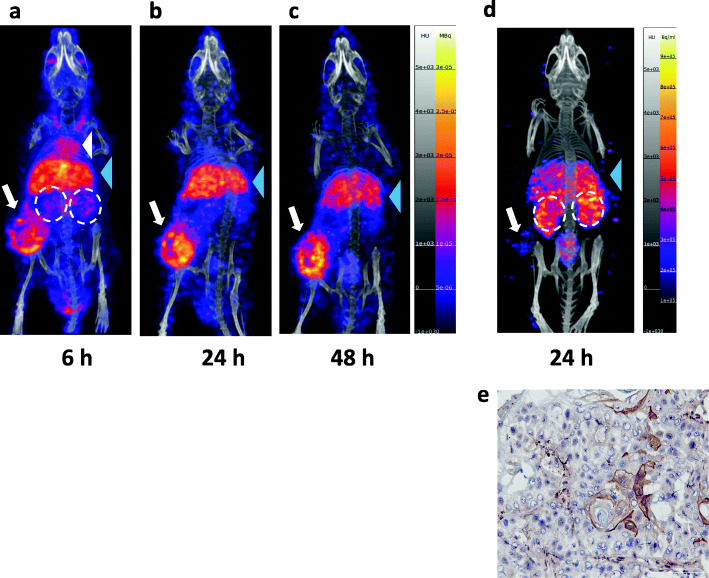
Posterior whole-body coronal microSPECT/CT images of a NRG mouse bearing s.c. implanted HNSCC PDX at **a.** 6 h, **b.** 24 h or **c.** 48 h p.i. of [^177^Lu]Lu-DOTA-panitumumab F(ab´)_2_ or **d.** 24 h p.i. of irrelevant [^177^Lu]Lu-DOTA-trastuzumab F(ab´)_2_. The tumour is shown by the white arrow. At 6 h p.i., the mediastinum (blood pool; white arrowhead), liver (blue arrowhead) and kidneys (broken white circles) were visualized, while at 24 and 48 h p.i., the liver and kidneys were the main normal organs visualized. **e.** Immunohistochemical staining of the excised tumour demonstrated very low HER2 expression

MicroPET/CT images were acquired at 6, 24 and 48 h p.i. of [^64^Cu]Cu-DOTA-panitumumab F(ab´)_2_ in NRG mice with s.c. HNSCC PDX (Fig. 3a-c). Tumours were visualized at all time points. Blood pool activity (mediastinal uptake) was observed at 6 h p.i but was not visible at 24 and 48 h p.i. Liver was the only normal organ visualized at all-time points but decreased at 24 and 48 h p.i. Kidneys were modestly visualized at 6 h p.i. but were not visible at 24 and 48 h p.i. IHC staining of excised tumours showed strong EGFR positivity (Fig. 3d). For comparison, microSPECT/CT images of NRG mice bearing the same s.c. HNSCC PDX were obtained at 6, 24 and 48 h p.i. of [^177^Lu]Lu-DOTA-panitumumab F(ab´)_2_ (Fig. 4a-c). Normal organ uptake of [^177^Lu]Lu-DOTA-panitumumab F(ab´)_2_ was similar to [^64^Cu]Cu-DOTA-panitumumab F(ab´)_2_. At 6 h p.i., blood pool activity (mediastinum) and uptake into the liver were observed. Blood pool activity decreased greatly at 24 and 48 h p.i. and liver uptake diminished slightly. The tumour was visualized at all time points. MicroSPECT/CT images of NRG mice bearing the same s.c. HNSCC PDX were obtained at 24 h p.i. of [^177^Lu]Lu-DOTA-trastuzumab F(ab´)_2_ (Fig. 4d). Normal organ uptake of [^177^Lu]Lu-DOTA-trastuzumab F(ab´)_2_ was similar to [^177^Lu]Lu-DOTA-panitumumab F(ab´)_2_ at 24 h p.i. but with higher kidney uptake. Tumours were not well-visualized by imaging with [^177^Lu]Lu-DOTA-trastuzumab F(ab´)_2_ in agreement with very low HER2 expression determined by IHC staining (Fig. 4e).

### Biodistribution (BOD) studies

The tumour and normal tissue BOD of [^64^Cu]Cu-DOTA-panitumumab F(ab´)_2_ or [^177^Lu]Lu-DOTA-panitumumab F(ab´)_2_ at 6, 24 or 48 h p.i. are shown in Table 1. Tumour uptake of [^64^Cu]Cu-DOTA-panitumumab F(ab´)_2_ was 9.3 ± 1.0%ID/g at 6 h p.i., but increased to 14.9 ± 1.1%ID/g at 24 h p.i., then declined to 9.8 ± 1.3%ID/g at 48 h p.i. Similarly, tumour uptake of [^177^Lu]Lu-DOTA-panitumumab F(ab´)_2_ was 10.4 ± 2.0%ID/g at 6 h p.i., increased to 18.0 ± 0.4%ID/g at 24 h p.i., then declined to 15.3 ± 2.6%ID/g at 48 h p.i. There were no significant differences in tumour uptake of [^64^Cu]Cu-DOTA-panitumumab F(ab´)_2_ compared to [^177^Lu]Lu-DOTA-panitumumab F(ab´)_2_ at any time points. Activity in the blood for mice injected with [^64^Cu]Cu-DOTA-panitumumab F(ab´)_2_ decreased from 10.6 ± 0.6%ID/g at 6 h p.i. to 4.8 ± 0.3%ID/g at 24 h p.i., then to 1.9 ± 0.1%ID/g at 48 h p.i. For mice injected with [^177^Lu]Lu-DOTA-panitumumab F(ab´)_2_, blood activity decreased from 15.3 ± 2.0%ID/g at 6 h p.i. to 4.5 ± 0.1%ID/g at 24 h p.i. then to 1.1 ± 0.2%ID/g at 48 h p.i. Blood activity was not significantly lower for [^64^Cu]Cu-DOTA-panitumumab F(ab´)_2_ than [^177^Lu]Lu-DOTA-panitumumab F(ab´)_2_ at 6 h p.i. but was significantly higher (*P* = 0.03) at 48 h p.i.. The tumour/blood (T/B) ratio for [^64^Cu]Cu-DOTA-panitumumab F(ab´)_2_ increased from 0.9 ± 0.1 at 6 h p.i. to 3.1 ± 0.1 at 24 h p.i. then to 5.1 ± 0.8 at 48 h p.i. The T/B ratio for [^177^Lu]Lu-DOTA-panitumumab F(ab´)_2_ increased from 0.7 ± 0.1 at 6 h p.i. to 4.0 ± 0.1 at 24 h p.i. then to 14.7 ± 2.8 at 48 h p.i. The T/B ratio was significantly greater for [^177^Lu]Lu-DOTA-panitumumab F(ab´)_2_ at 48 h p.i. than [^64^Cu]Cu-DOTA-panitumumab F(ab´)_2_ (*P* = 0.03) but not at 6 or 24 h p.i. At 6 h p.i., there was low uptake in normal tissues in mice injected with [^64^Cu]Cu-DOTA-panitumumab F(ab´)_2_ and normal tissue uptake was not significantly different than [^177^Lu]Lu-DOTA-panitumumab F(ab´)_2_. (Table 1). At 24 h p.i., there was insignificantly lower uptake in the lungs in mice injected with [^177^Lu]Lu-DOTA-panitumumab F(ab´)_2_ than [^64^Cu]Cu-DOTA-panitumumab F(ab´)_2_. Liver uptake was insignificantly higher for [^177^Lu]Lu-DOTA-panitumumab F(ab´)_2_. At 48 h p.i., there was significantly higher uptake in the blood and several normal tissues including the liver for [^64^Cu]Cu-DOTA-panitumumab F(ab´)_2_ than [^177^Lu]Lu-DOTA-panitumumab F(ab´)_2_ (*P* < 0.05). However, skin and muscle uptake were significantly higher for [^177^Lu]Lu-DOTA-panitumumab F(ab´)_2_. The tumour and normal tissue BOD of irrelevant [^177^Lu]Lu-DOTA-trastuzumab F(ab´)_2_ is also shown in Table 1. Tumour uptake of [^177^Lu]Lu-DOTA-trastuzumab F(ab´)_2_ at 24 h p.i. was 2.6 ± 0.5%ID/g, which was 5.7 times (*P* = 0.04) and 6.9 times (*P* = 0.04) significantly lower than [^64^Cu]Cu-DOTA-panitumumab F(ab´)_2_ and [^177^Lu]Lu-DOTA-panitumumab F(ab´)_2_, respectively. Kidney uptake of [^177^Lu]Lu-DOTA-trastuzumab F(ab´)_2_ (27.4 ± 2.7%ID/g) was significantly higher than [^64^Cu]Cu-DOTA-panitumumab F(ab´)_2_ (4.7 ± 0.5%ID/g; *P* = 0.04) or [^177^Lu]Lu-DOTA-panitumumab F(ab´)_2_ (5.9 ± 0.0%ID/g; *P* = 0.04). Liver uptake of [^177^Lu]Lu-DOTA-trastuzumab F(ab´)_2_ (8.0 ± 1.0%ID/g) was significantly lower than [^177^Lu]Lu-DOTA-panitumumab F(ab´)_2_ (12.1 ± 1.1%ID/g; *P* = 0.04) but not significantly different than [^64^Cu]Cu-DOTA-panitumumab F(ab´)_2_ (7.5 ± 1.0%ID/g; *P* > 0.05). A comparison of the tumour and liver uptake of [^64^Cu]Cu-DOTA-panitumumab F(ab´)_2_ measured by ROI analysis of microPET/CT images or BOD studies is shown in Table 2. There were no significant differences in the measured tumour uptake but the ROI analysis modestly overestimated the liver uptake of [^64^Cu]Cu-DOTA-panitumumab F(ab´)_2_ at 48 h p.i.

**Table 1:** Tumour and normal tissue biodistribution (BOD) of radioimmunoconjugates in NRG mice with s.c. HNSCC primary xenografts ^a^

	[^**64**^Cu]Cu-DOTA-panitumumab F(ab´)_**2**_							[^**177**^Lu]Lu-DOTA-panitumumab F(ab´)_**2**_				[^**177**^Lu]Lu-DOTA-trastuzumab F(ab’)_**2**_
						Percent injected dose/g (%ID/g)						
**Time p.i.:**	**6 h**	***P-*** **value**^**b**^	**24 h**	***P-*value**^**b**^	***P-*value**^**c**^	**48 h**	***P*-value**^**b**^	**6 h**	**24 h**	***P-*value**^**c**^	**48 h**	**24 h**
Heart	3.5 ± 0.5	n.s.	2.8 ± 0.3	n.s.	0.04	1.5 ± 0.1	n.s.	4.4 ± 0.2	2.9 ± 0.05	0.04	1.5 ± 0.5	1.3 ± 0.2
Lungs	3.9 ± 0.2	n.s.	3.4 ± 0.2	n.s.	0.04	2.4 ± 0.0	0.03	5.2 ± 0.6	2.1 ± 0.3	0.04	1.3 ± 0.0	1. 1 ± 0.1
Stomach	1.5 ± 0.1	n.s.	1.8 ± 0.1	n.s.	0.04	1.5 ± 0.1	0.03	1.8 ± 0.1	1.4 ± 0.0	0.04	0.8 ± 0.1	0.4 ± 0.05
Pancreas	1.0 ± 0.1	n.s.	1.2 ± 0.2	n.s.	n.s.	1.2 ± 0.2	0.03	1.2 ± 0.2	1.0 ± 0.1	n.s.	0.6 ± 0.1	0.5 ± 0.2
Intestine	1.2 ± 0.0	n.s.	1.7 ± 0.1	n.s.	0.04	1.5 ± 0.1	0.03	1.9 ± 0.1	1.3 ± 0.1	0.04	1.0 ± 0.1	0.4 ± 0.07
Spleen	2.4 ± 0.1	n.s.	3.5 ± 0.3	n.s.	n.s.	4.0 ± 0.7	n.s.	3.3 ± 0.1	4.3 ± 0.6	n.s.	3.8 ± 0.4	5.9 ± 1.4
Liver	8.4 ± 0.8	n.s.	7.5 ± 1.0	n.s.	n.s.	6.2 ± 0.1	0.03	10.1 ± 1.6	12.1 ± 1.1	0.04	11.0 ± 0.9	8.0 ± 1.0
Kidneys	5.5 ± 0.2	n.s.	4.7 ± 0.5	n.s.	0.04	3.0 ± 0.2	n.s.	8.1 ± 0.5	5.9 ± 0.0	0.04	4.1 ± 0.4	27.4 ± 2.7
Bone	1.0 ± 0.3	n.s.	0.6 ± 0.1	n.s.	n.s.	0.6 ± 0.1	n.s.	1.2 ± 0.1	1.8 ± 0.7	n.s.	0.6 ± 0.0	2.6 ± 1.6
Skin	1.0 ± 0.3	n.s.	2.7 ± 1.2	n.s.	n.s.	1.0 ± 0.1	0.03	2.0 ± 0.3	2.2 ± 0.7	n.s.	1.6 ± 0.1	0.9 ± 0.2
Muscle	0.6 ± 0.1	n.s.	0.4 ± 0.1	n.s.	n.s.	0.4 ± 0.0	0.03	0.7 ± 0.1	0.6 ± 0.1	n.s.	0.5 ± 0.0	0.3 ± 0.07
Blood	10.6 ± 0.6	n.s.	4.8 ± 0.3	n.s.	0.04	1.9 ± 0.1	0.03	15.3 ± 0.6	4.5 ± 0.1	0.04	1.1 ± 0.2	1.3 ± 0.3
Tumour	9.3 ± 1.0	n.s.	14.9 ± 1.1	n.s.	0.04	9.8 ± 1.3	n.s.	10.4 ± 2.0	18.0 ± 0.4	0.04	15.3 ± 2.6	2.6 ± 0.5
T/B Ratio ^d^	0.9 ± 0.1	n.s.	3.1 ± 0.1	n.s.	n.s.	5.1 ± 0.8	0.03	0.7 ± 0.1	4.0 ± 0.1	n.s.	14.7 ± 2.8	2.2 ± 0.6

^a^[^64^Cu]Cu-DOTA-panitumumab F(ab´)_2_ (5.5–14.0 MBq; 50 μg) or [^177^Lu]Lu-DOTA-panitumumab F(ab´)_2_ (6.5 MBq; 50 μg) were injected i.v. (tail vein) in groups (*n* = 3–4) of tumour-bearing NRG mice

^b^*P*-value for comparison of [^64^Cu]Cu-DOTA-panitumumab F(ab´)_2_ and [^177^Lu]Lu-DOTA-panitumumab F(ab´)_2_ at the same time point (6, 24 or 48 h) post-injection using a Mann-Whitney U test

^c^*P*-value for comparison of [^64^Cu]Cu-DOTA-panitumumab F(ab´)_2_ or [^177^Lu]Lu-DOTA-panitumumab F(ab´)_2_ to [^177^Lu]Lu-DOTA-trastuzumab F(ab´)_2_ at 24 h p.i. using a Mann-Whitney U test

^d^T/B: tumour:blood

**Table 2:** Comparison of the tumour and liver uptake of radioimmunoconjugates (RICs) estimated by region of interest (ROI) analysis of PET images and biodistribution (BOD) studies

Percent injected dose/g (mean ± SEM)						
	Tumour			Liver		
**Time p.i.**	**PET**^**a**^	**BOD**^**b**^	***P*-value**^**c**^	**PET**^**a**^	**BOD**^**b**^	***P*-value**^**c**^
6 h	8.8 ± 2.3	9.3 ± 1.0	n.s.	11.2 ± 0.5	8.4 ± 0.8	n.s.
24 h	10.3 ± 1.3	14.9 ± 1.1	n.s.	9.5 ± 0.2	7.5 ± 1.0	n.s.
48 h	10.9 ± 1.1	9.8 ± 1.3	n.s.	7.2 ± 0.3	6.2 ± 0.1	0.04

^a^Uptake of [^64^Cu]Cu-DOTA-panitumumab Fab´)_2_ estimated by ROI analysis of PET images expressed in percent injected dose/g (%ID/g; mean ± SEM, *n* = 4)

^b^Uptake of [^64^Cu]Cu-DOTA-panitumumab F(ab´)_2_ estimated by BOD analysis expressed in %ID/g (mean ± SEM, n = 4)

^c^Comparison of ROI and BOD analysis by a Mann-Whitney U test (*P* < 0.05)

### Tumour and normal organ dosimetry

The BOD of [^64^Cu]Cu-DOTA-panitumumab F(ab´)_2_ or [^177^Lu]Lu-DOTA-panitumumab F(ab´)_2_ in NRG mice with s.c. HNSCC PDXs was used to estimate the radiation equivalent doses (Table 3). In addition, doses for [^177^Lu]Lu-DOTA-panitumumab F(ab´)_2_ were estimated from the BOD of [^64^Cu]Cu-DOTA-panitumumab F(ab´)_2_ for comparison with those estimated from the BOD of [^177^Lu]Lu-DOTA-panitumumab F(ab´)_2_. We first calculated the doses from [^64^Cu]Cu-DOTA-panitumumab F(ab´)_2_ in NRG tumour-bearing mice. The liver was the normal organ which received the highest dose (0.10 ± 0.02 Sv/MBq) followed by the kidneys (0.07 ± 0.01 Sv/MBq), lungs (0.05 ± 0.01 Sv/MBq), spleen (0.04 ± 0.01 Sv/MBq), stomach (0.03 ± 0.01 Sv/MBq), heart (0.03 ± 0.01 Sv/MBq) and intestine (0.02 ± 0.00 Sv/MBq). The tumour received the highest dose (0.14 ± 0.03 Sv/MBq). The whole body equivalent dose was 0.05 ± 0.00 Sv/MBq. The doses for [^177^Lu]Lu-DOTA-panitumumab F(ab´)_2_ exhibited a similar trend but were higher. Among the normal organs, the highest doses were in the liver (1.82 ± 0.14 Sv/MBq), kidneys (0.75 ± 0.08 Sv/MBq), spleen (0.66 ± 0.18 Sv/MBq), lungs (0.38 ± 0.04 Sv/MBq), heart (0.32 ± 0.13 mSv/MBq), stomach (0.27 ± 0.04 mSv/MBq), intestines (0.21 ± 0.05 mSv/MBq) and pancreas (0.16 ± 0.04 Sv/MBq). The tumour dose and whole body dose were 2.5 ± 0.8 Sv/MBq and 0.34 ± 0.02 Sv/MBq, respectively which were about 7 times higher than those for [^64^Cu]Cu-DOTA-panitumumab F(ab´)_2_.

**Table 3:** Radiation equivalent doses in the tumour and normal organs for radioimmunoconjugates in NRG mice with s.c. HNSCC primary xenografts ^a^

	Radiation equivalent dose (Sv/MBq)			
Organ	[^**64**^Cu]Cu-DOTA-panitumumab F(ab´)_**2**_^**b**^	[^**177**^Lu]Lu-DOTA-panitumumab F(ab´)_**2**_^**c**^	[^**177**^Lu]Lu-DOTA-panitumumab F(ab´)_**2**_^**d**^	Comparison of doses for ^**177**^Lu calculated from ^**64**^Cu vs. ^**177**^Lu biodistribution (BOD) (***P***-value)
Heart	0.03 ± 0.01	0.35 ± 0.06	0.32 ± 0.13	n.s.
Lungs	0.05 ± 0.01	0.51 ± 0.05	0.38 ± 0.04	n.s.
Liver	0.10 ± 0.02	1.22 ± 0.13	1.82 ± 0.14	0.03
Spleen	0.04 ± 0.01	0.71 ± 0.20	0.66 ± 0.18	n.s.
Pancreas	0.02 ± 0.01	0.26 ± 0.08	0.16 ± 0.04	n.s.
Stomach	0.03 ± 0.01	0.33 ± 0.03	0.27 ± 0.04	n.s.
Intestines	0.02 ± 0.00	0.31 ± 0.05	0.21 ± 0.05	n.s.
Kidneys	0.07 ± 0.01	0.63 ± 0.09	0.75 ± 0.08	n.s.
Tumour ^e^	0.14 ± 0.03	2.00 ± 0.60	2.50 ± 0.80	n.s.
Whole Body	0.05 ± 0.00	0.26 ± 0.02	0.34 ± 0.02	0.05

^a^Equivalent doses (D) were calculatd as D = Ã_s_ × S × W_R_, where Ã_s_ is the time-integrated activity in source organs obtained from BOD studies and S are the Snyder values for mice (Bitar et al., 2007; Xie and Zaidi, 2013) and W_R_ is the radiation weighing factor

^b^Equivalent doses for [^64^Cu]Cu-DOTA-panitumumab F(ab´)_2_

^c^Equivalent doses for [^177^Lu]Lu-DOTA-panitumumab F(ab´)_2_ calculated based on the time-integrated activity for [^64^Cu]Cu-DOTA-panitumumab F(ab´)_2_ and using S-values for ^177^Lu

^d^Equivalent doses for [^177^Lu]Lu-DOTA-panitumumab F(ab´)_2_ calculated based on the time-integrated activity for [^177^Lu]Lu-DOTA-panitumumab F(ab´)_2_ and using S-values for ^177^Lu

^e^Self-equivalent doses were estimated using the sphere model in OLINDA/EXM software based on the measured tumour volume for ^64^Cu or ^177^Lu

Estimates of the radiation equivalent doses for [^177^Lu]Lu-DOTA-panitumumab F(ab´)_2_ based on the BOD of [^64^Cu]Cu-DOTA-panitumumab F(ab´)_2_ were made for comparison (Table 3). The highest doses were received by the liver (1.22 ± 0.13 Sv/MBq), spleen (0.71 ± 0.20 Sv/MBq), kidneys (0.63 ± 0.09 Sv/MBq), lungs (0.51 ± 0.05 Sv/MBq), heart (0.35 ± 0.06 Sv/MBq), stomach (0.33 ± 0.03 Sv/MBq), intestines (0.31 ± 0.05 Sv/MBq) and pancreas (0.26 ± 0.08 Sv/MBq). The tumour dose was 2.00 ± 0.60 Sv/MBq and the whole-body dose was 0.26 ± 0.02 Sv/MBq. The doses estimated for [^177^Lu]Lu-DOTA-panitumumab F(ab´)_2_ based on the BOD of [^64^Cu]Cu-DOTA-panitumumab F(ab´)_2_ were not significantly different than those estimated from the BOD of [^177^Lu]Lu-DOTA-panitumumab F(ab´)_2_ except for the liver (*P* = 0.03) and whole body (*P* = 0.05).

The tumour and liver uptake estimated by ROI analysis of microPET/CT images were used to estimate the doses for [^64^Cu]Cu-DOTA-panitumumab F(ab´)_2_ or [^177^Lu]Lu-DOTA-panitumumab F(ab´)_2_ and were compared to doses calculated from BOD studies of [^64^Cu]Cu-DOTA-panitumumab F(ab´)_2_ or [^177^Lu]Lu-DOTA-panitumumab F(ab´)_2_. ROI-based estimates of the doses for [^64^Cu]Cu-DOTA-panitumumab F(ab´)_2_ in the tumour (0.12 ± 0.04 Sv/MBq) and liver (0.14 ± 0.02 Sv/MBq) did not differ significantly from those based on BOD studies of [^64^Cu]Cu-DOTA-panitumumab F(ab´)_2_ (0.14 ± 0.02 Sv/MBq, 0.10 ± 0.02 Sv/MBq, respectively; Supplementary Information Table S1). Similarly, ROI-based estimates of the doses for [^177^Lu]Lu-DOTA-panitumumab F(ab´)_2_ in the tumour (2.3 ± 0.4 Sv/MBq) and liver (1.45 ± 0.17 Sv/MBq) were not significantly different than those based on BOD studies of [^64^Cu]Cu-DOTA-panitumumab F(ab´)_2_ (2.0 ± 0.6 Sv/MBq and 1.22 ± 0.13 Sv/MBq espectively) or [^177^Lu]Lu-DOTA-panitumumab F(ab´)_2_ (2.5 ± 0.8 Sv/MBq and 1.82 ± 0.14 Sv/MBq, respectively; Table 4).

**Table 4:** Radiation equivalent doses in the tumour and liver in NRG mice with s.c. HNSCC primary xenografts for [^177^Lu]Lu-DOTA-panitumumab F(ab´)_2_ based on ROI analysis of biodistribution (BOD)

	Radiation equivalent dose ^**a**^ (Sv/MBq)				
Organ	^**64**^Cu/PET ^**b**^	^**64**^Cu/BOD ^**c**^	Comparison of doses for ^**177**^Lu estimated from ^**64**^Cu/PET ^**b**^ or ^**64**^Cu/BOD ^**c**^ (***P***-value)	^**177**^Lu/BOD ^**d**^	Comparison of doses for ^**177**^Lu estimated from ^**64**^Cu/PET ^**b**^ or ^**177**^Lu/BOD ^**d**^ (***P***-value)
Liver	1.45 ± 0.17	1.22 ± 0.13	n.s.	1.82 ± 0.14	n.s.
Tumour ^e^	2.30 ± 0.40	2.00 ± 0.60	n.s.	2.50 ± 0.80	n.s.

^a^The equivalent doses (D) were calculated as D = Ã_s_ × S × W_R_, where Ã_s_ is the time-integrated activity in source organs and S are the Snyder values for ^177^Lu for mice (Bitar et al., 2007; Xie and Zaidi, 2013) and W_R_ is the radiation weighing factor

^b^The time-integrated radioactivity was calculated based on microPET/CT studies of NRG mice injected i.v. (tail vein) with [^64^Cu]Cu-DOTA-panitumumab F(ab´)_2_

^c^The time-integrated radioactivity was calculated based on the BOD studies of NRG mice injected i.v. (tail vein) with [^64^Cu]Cu-DOTA-panitumumab F(ab´)_2_

^d^The time-integrated radioactivity was calculated based on the BOD studies of NRG mice injected i.v. (tail vein) with [^177^Lu]Lu-DOTA-panitumumab F(ab´)_2_

^e^Estimated using the sphere model in OLINDA/EXM software based on the measured tumour mass (Stabin et al., 2005)

The radiation equivalent doses in an adult human female with a hypothetical 2 cm diameter spherical tumour in the neck from administration of [^64^Cu]Cu-DOTA-panitumumab F(ab´)_2_ or [^177^Lu]Lu-DOTA-panitumumab F(ab´)_2_ were projected based on BOD studies or ROI analysis of PET images in mice with s.c. HNSCC PDX (Supplementary Table S2). Using the BOD data, [^64^Cu]Cu-DOTA-panitumumab F(ab´)_2_ deposited the highest doses in the liver (0.065 ± 0.012 mSv/MBq), kidneys (0.043 ± 0.006 mSv/MBq), intestines (0.037 ± 0.002 mSv/MBq) and tumour (0.092 ± 0.017 mSv/MBq). The whole body dose was 0.030 ± 0.002 mSv/MBq. Doses based on ROI analysis of the images were estimated for the liver (0.080 ± 0.009 mSv/MBq) and tumour (0.065 ± 0.016 mSv/MBq). These were similar to those estimated using the BOD studies. Using the BOD, [^177^Lu]Lu-DOTA-panitumumab F(ab´)_2_ deposited the highest doses in the liver (1.05 ± 0.08 mSv/MBq), kidneys (0.44 ± 0.04 mSv/MBq), spleen (0.39 ± 0.11 mSv/MBq) and tumour (1.47 ± 0.46 mSv/MBq). The whole body dose was 0.22 ± 0.02 mSv/MBq. The doses deposited by [^177^Lu]Lu-DOTA-panitumumab F(ab´)_2_ were higher than those from [^64^Cu]Cu-DOTA-panitumumab F(ab´)_2_. The doses for [^177^Lu]Lu-DOTA-panitumumab F(ab´)_2_ based on BOD or ROI analysis of the PET images of [^64^Cu]Cu-DOTA-panitumumab F(ab´)_2_ were similar to those estimated from the BOD of [^177^Lu]Lu-DOTA-panitumumab F(ab´)_2_ except for the liver (0.70 ± 0.07 mSv/MBq and 0.83 ± 0.10 mSv/MBq for BOD and ROI analysis, respectively) and tumour (1.10 ± 0.32 mSv/MBq and 1.14 ± 0.22 mSv/MBq for BOD and ROI analysis, respectively) which were modestly underestimated (Supplementary Table S2).

## Discussion

We report here for the first time radiation equivalent dose predictions for [^177^Lu]Lu-DOTA-panitumumab F(ab´)_2_ based on BOD studies and ROI analysis of microPET/CT images in NRG mice with s.c. HNSCC PDX tumours after i.v. administration of [^64^Cu]Cu-DOTA-panitumumab F(ab´)_2_. Our results indicate that a PET theranostic strategy employing [^64^Cu]Cu-DOTA-panitumumab F(ab´)_2_ to image HNSCC tumours and predict the doses to the tumour and normal organs from RIT with [^177^Lu]Lu-DOTA-panitumumab F(ab´)_2_ is feasible. The doses in the tumour and normal organs for [^177^Lu]Lu-DOTA-panitumumab F(ab´)_2_ that were estimated from the BOD studies of [^64^Cu]Cu-DOTA-panitumumab F(ab´)_2_ were not significantly different than those estimated directly from the BOD of [^177^Lu]Lu-DOTA-panitumumab F(ab´)_2_ except for the liver and tumour, which were modestly underestimated by [^64^Cu]Cu-DOTA-panitumumab F(ab´)_2_ (Tables 3 and 4). To our knowledge, our report is also the first to describe microPET/CT of HNSCC PDX with [^64^Cu]Cu-DOTA-panitumumab F(ab´)_2_ (Fig. 3) or microSPECT/CT of these tumours with [^177^Lu]Lu-DOTA-panitumumab F(ab´)_2_ (Fig. 4).

F(ab´)_2_ were produced from panitumumab IgG in high purity (Fig. 1) and were conjugated to DOTA for complexing ^64^Cu for PET or ^177^Lu for RIT. The stability constant of DOTA complexed to Cu^2+^ (log K = 23.3) (Kubíček et al., 2018) is lower than Lu^3+^ (log K = 26.7) (Majkowska and Bilewicz, 2007) but both complexes are stable. DOTA-F(ab´)_2_ efficiently chelated ^64^Cu or ^177^Lu achieving a RCP of 95.5 ± 2.1% and 96.7 ± 3.5%, respectively after incubation for 1 h at 42 °C. The results of a saturation radioligand binding assay revealed high affinity binding (K_d_ = 2.9 ± 0.7 × 10^− 9^ mol/L) of [^177^Lu]Lu-DOTA-panitumumab F(ab´)_2_ to EGFR-positive MDA-MB-468 human breast cancer cells (Fig. 2). The K_d_ for [^177^Lu]Lu-DOTA-panitumumab F(ab′)_2_ was 3-fold higher than we previously reported for [^177^Lu]Lu-DOTA-panitumumab IgG (K_d_ = 1.0 ± 0.4 10^− 9^ mol/L) (Aghevlian et al., 2018). We did not measure the EGFR binding affinity of [^64^Cu]Cu-DOTA-panitumumab F(ab´)_2_ but substitution of ^64^Cu for ^177^Lu is not expected to change the affinity. Irrelevant F(ab´)_2_ were produced in high purity from trastuzumab IgG (Fig. S1), conjugated to DOTA and labeled with ^177^Lu to high RCP (> 97%) to provide specificity control RICs for imaging and BOD studies.

MicroPET/CT visualized HNSCC PDX in NRG mice at 6 h p.i. and especially at 24 h and 48 h p.i. of [^64^Cu]Cu-DOTA-panitumumab F(ab´)_2_ (Fig. 3a-c). These PDX were EGFR positive by IHC staining (Fig. 3d). HNSCC PDX were also imaged by microSPECT/CT at 6, 24 or 48 h p.i. of [^177^Lu]Lu-DOTA-panitumumab F(ab′)_2_ (Fig. 4a-c) but these tumours were not visualized at 24 h p.i. of irrelevant [^177^Lu]Lu-DOTA-trastuzumab F(ab′)_2_ (Fig. 4d). IHC staining of the PDX showed very low HER2 expression (Fig. 4e). BOD studies at 24 h p.i. demonstrated higher tumour uptake and T/B ratios for [^64^Cu]Cu-DOTA-panitumumab F(ab´)_2_ and [^177^Lu]Lu-DOTA-panitumumab F(ab′)_2_ than [^177^Lu]Lu-DOTA-trastuzumab F(ab′)_2_. These results indicated that tumour imaging with [^64^Cu]Cu-DOTA-panitumumab F(ab´)_2_ or [^177^Lu]Lu-DOTA-panitumumab F(ab′)_2_ was EGFR-mediated. There was higher kidney uptake of [^64^Cu]Cu-DOTA-trastuzumab F(ab')_2_ (27.4 ± 2.7%ID/g) than [^64^Cu]Cu-DOTA-panitumumab F(ab')_2_ (8.1 ± 0.5%ID/g at 24 h p.i. (Table 1). Based on the amino acid sequences obtained from the Protein Data Bank in Europe (Anonymous, 2021b) we estimated the pI of these F(ab')_2_ by entering the sequences into the Compute pI/Mw tool on ExPASy (Anonymous, 2021a). The pI of trastuzumab F(ab')_2_ and panitumumab F(ab')_2_ were estimated to be 8.58 and 6.71, respectively. At physiological pH = 7.4, trastuzumab F(ab')_2_ would be cationic and panitumumab F(ab')_2_ would be slightly anionic. The cationic charge of some mAb fragments is known to promote kidney uptake (Behr et al., 1995) which may explain the higher renal accumulation of [^64^Cu]Cu-DOTA-trastuzumab F(ab')_2_. Although PDX in NRG mice were imaged with [^177^Lu]Lu-DOTA-panitumumab F(ab′)_2_, SPECT has poor sensitivity due to the low abundance of the γ-photons emitted by ^177^Lu [Eγ = 113 keV (6.4%) and Eγ = 208 keV(11%)]. In addition, the dosimetry of ^177^Lu is not favourable for imaging. The whole body equivalent dose for [^177^Lu]Lu-DOTA-panitumumab F(ab′)_2_ in NRG mice (0.34 ± 0.02 Sv/MBq) was 7-fold higher than [^64^Cu]Cu-DOTA-panitumumab F(ab´)_2_ (0.05 ± 0.00 Sv/MBq; Table 3). The whole body dose projected in humans was 3-fold higher for [^177^Lu]Lu-DOTA-panitumumab F(ab′)_2_ than [^64^Cu]Cu-DOTA-panitumumab F(ab´)_2_ (0.15 ± 0.01 vs. 0.030 ± 0.002 mSv/MBq, respectively; Supplementary Information Table S2).

A PET theranostic strategy relies on accurate dose predictions for [^177^Lu]Lu-DOTA-panitumumab F(ab′)_2_ based on the BOD of [^64^Cu]Cu-DOTA-panitumumab F(ab´)_2_. BOD studies in NRG mice with s.c. HNSCC PDX at 6 or 24 p.i. showed similar uptake of these two RICs (Table 1) with only modest but significant differences in tissue localization at 48 h p.i. between [^64^Cu]Cu-DOTA-panitumumab F(ab´)_2_ and [^177^Lu]Lu-DOTA-panitumumab F(ab′)_2_. At 6 h p.i., uptake of [^64^Cu]Cu-DOTA-panitumumab F(ab´)_2_ was lower than [^177^Lu]Lu-DOTA-panitumumab F(ab′)_2_ in the intestine, spleen, kidneys and skin but higher in the blood. At 24 h p.i., lung uptake of [^64^Cu]Cu-DOTA-panitumumab F(ab´)_2_ was higher than [^177^Lu]Lu-DOTA-panitumumab F(ab′)_2_ but liver uptake was lower. At 48 h p.i., the uptake of [^64^Cu]Cu-DOTA-panitumumab F(ab´)_2_ was higher than [^177^Lu]Lu-DOTA-panitumumab F(ab′)_2_ in the lungs, stomach, pancreas, intestines and blood but lower in the liver and skin (*P* < 0.05). These modest differences in BOD may be due to experimental variability in tissue uptake in BOD studies. It is possible that the lower stability constant of DOTA for complexing Cu^2+^ (log K = 23.3) (Kubíček et al., 2018) vs. Lu^3+^ (log K = 26.7) may cause some differences in BOD between the two RICs (Majkowska and Bilewicz, 2007). The lower uptake of [^64^Cu]Cu-DOTA-panitumumab F(ab´)_2_ in the liver than [^177^Lu]Lu-DOTA-panitumumab F(ab′)_2_ at 24 h p.i. was unexpected, since release of ^64^Cu from DOTA should increase liver uptake (Hausner et al., 2009). There were no significant differences in tumour uptake of [^64^Cu]Cu-DOTA-panitumumab F(ab´)_2_ and [^177^Lu]Lu-DOTA-panitumumab F(ab′)_2_ at any time point. In patients, the tumour and normal organ uptake of [^64^Cu]Cu-DOTA-panitumumab F(ab´)_2_ would be measured by ROI analysis to predict doses from [^177^Lu]Lu-DOTA-panitumumab F(ab′)_2_. Thus, we compared the uptake of the RICs into the tumour and liver measured by ROI analysis of microPET/CT images with that measured in BOD studies (Supplementary Information Table S1). There were no significant differences in quantification of tumour uptake by these two methods, but ROI analysis overestimated the liver uptake. This may be due to imprecise delineation of the liver ROI resulting in some inaccuracy in measuring liver radioactivity. Manual segmentation in ROI analysis on PET images is subject to variability in delineation, contributing to differences in the measuring the radioactivity (Foster et al., 2014).

The radiation equivalent doses in the tumour and normal organs were calculated as D = Ã_s_ × S × W_R_, where Ã_s_ is the time-integrated activity in the tumour or normal source organs and S are the Snyder values for mice (Bitar et al., 2007; Xie and Zaidi, 2013) and W_R_ is the radiation weighing factor. The total Ã_s_ was calculated by summing the area-under-the-curve in source organs from time zero to 48 h p.i. (AUC_0–48 h;_ Bq × sec) and the Ã_48 h – ∞_ from 48 h p.i. to infinity (Bq × sec). The calculation of Ã_s_ tends to minimize variability in BOD between [^64^Cu]Cu-DOTA-panitumumab F(ab´)_2_ and [^177^Lu]Lu-DOTA-panitumumab F(ab′)_2_ at individual time points. Consequently, we found no significant differences in the doses estimated for [^177^Lu]Lu-DOTA-panitumumab F(ab′)_2_ based on the BOD of [^64^Cu]Cu-DOTA-panitumumab F(ab´)_2_ compared to those based on the BOD of [^177^Lu]Lu-DOTA-panitumumab F(ab′)_2_ except for the liver and whole body, which were modestly underestimated by [^64^Cu]Cu-DOTA-panitumumab F(ab´)_2_ (Table 3). The higher absorbed dose in the liver for [^177^Lu]Lu-DOTA-panitumumab F(ab’)_2_ than [^64^Cu]Cu-DOTA-panitumumab F(ab')_2_ is due to the higher liver uptake in BOD studies. The reason for the higher liver uptake of [^177^Lu]Lu-DOTA-panitumumab F(ab')_2_ is not known. However, DOTA forms higher coordination number and more stable complexes with ^177^Lu (coordination number = 8; logK = 23.1–29.2) than ^64^Cu (coordination number = 6; logK = 22.2–22.7) (Viola-Villegas and Doyle, 2009) which may result in greater retention of ^177^Lu than ^64^Cu in the liver following hepatic uptake and metabolism of the RICs. ROI analysis of PET images with [^64^Cu]Cu-DOTA-panitumumab F(ab´)_2_ to estimate the doses in the tumour or liver yielded estimates for [^177^Lu]Lu-DOTA-panitumumab F(ab′)_2_ that were not significantly different than those based on the BOD of [^64^Cu]Cu-DOTA-panitumumab F(ab´)_2_ or [^177^Lu]Lu-DOTA-panitumumab F(ab′)_2_ (Table 4). The projected doses in a hypothetical 2 cm spherical tumour in the neck of a patient with HNSCC from [^177^Lu]Lu-DOTA-panitumumab F(ab′)_2_ was 1.10 ± 0.32 mSv/MBq based on the BOD in mice of [^64^Cu]Cu-DOTA-panitumumab F(ab´)_2_ and 1.47 ± 0.46 mSv/MBq based on [^177^Lu]Lu-DOTA-panitumumab F(ab′)_2_ (Supplementary Information Table S2). The whole body dose was 0.15 ± 0.01 mSv/MBq based on the BOD of [^64^Cu]Cu-DOTA-panitumumab F(ab´)_2_ and 0.22 ± 0.01 mSv/MBq based on the BOD of [^177^Lu]Lu-DOTA-panitumumab F(ab′)_2_. Fractionated doses of external radiation are used to treat HNSCC (2 Gy per cycle to a total of 70 Gy) (Hutchinson et al., 2020). The total dose required for effective treatment of malignancies with RIT is not well-defined (Macklis, 2004). Other factors such as the tumour growth inhibitory properties of the mAbs, bystander effects (Boyd et al., 2008) or a radiation-induced anti-tumour immune response (ie. abscopal effect) (Gorin et al., 2014) may contribute to the effectiveness of RIT. In addition, dose rates for RIT are exponentially decreasing and are much lower than external radiation which may affect tumour response at the same dose as external radiation (Fowler, 1990). The safe and effective administered amount of [^177^Lu]Lu-DOTA-panitumumab F(ab′)_2_ and the respective doses should be determined in a future clinical trial.

The liver accumulated [^177^Lu]Lu-DOTA-panitumumab F(ab′)_2_ (Fig. 4 and Table 1). The dose in the liver projected in humans was 0.70 ± 0.07 mSv/MBq based on the BOD of [^64^Cu]Cu-DOTA-panitumumab F(ab´)_2_ and 1.05 ± 0.08 mSv/Bq based on the BOD of [^177^Lu]Lu-DOTA-panitumumab F(ab′)_2_ (Supplementary Information Table S2). Preclinically, we found no increase in serum liver transaminases suggesting no liver toxicity in NRG mice with s.c. human pancreatic cancer xenografts treated with [^177^Lu]Lu-DOTA-panitumumab IgG (6 MBq; 50 μg), although the estimated dose in the liver was 6500 mSv (Aghevlian et al., 2020). Thus, dosimetry projections in humans suggest that RIT [^177^Lu]Lu-DOTA-trastuzumab F(ab′)_2_ may be safe and effective for treatment of HNSCC. However, our studies projected human doses based on the BOD of [^177^Lu]Lu-DOTA-panitumumab F(ab′)_2_ in NRG mice and panitumumab does not recognize mouse EGFR (Tabrizi et al., 2006), thus some differences in BOD of the RICs and normal organ doses may be found in patients. In addition, soluble EGFR has been detected in the plasma of patients with HNSCC (Polanska et al., 2016). Binding to soluble EGFR may alter the pharmacokinetics of the RICs and may promote uptake of these immune complexes in the liver and spleen. We did not measure soluble EGFR in these mouse PDX models and thus how soluble EGFR in the plasma affects the biodistribution and dose estimations for the RICs is not known.

## Conclusions

We conclude that a PET theranostic strategy employing [^64^Cu]Cu-DOTA-panitumumab F(ab´)_2_ to image HNSCC in patients and predict the radiation equivalent doses in a tumour and normal organs from RIT with [^177^Lu]Lu-DOTA-panitumumab F(ab′)_2_ is feasible. RIT with [^177^Lu]Lu-DOTA-panitumumab F(ab′)_2_ may be a promising approach to treatment of HNSCC due to frequent overexpression of EGFR.

## Supplementary Information

Additional file 1 **Fig. S1.** SDS PAGE analysis of trastuzumab IgG and F(ab′)_2_ under non-reducing (lanes 1, 2, respectively) or reducing (lanes 3, 4, respectively) conditions on a 7.5% Tris/Glycine mini-gel stained with Coomassie blue. **Table S1.** Estimated radiation equivalent doses in a tumour-bearing NRG mouse for [^64^Cu]Cu-DOTA-panitumumab F(ab´)_2_. **Table S2.** Projected radiation equivalent doses for a female human adult with a 2 cm tumour in the neck for [^64^Cu]Cu-DOTA-panitumumab F(ab´)_2_ or [^177^Lu]Lu-DOTA-panitumumab F(ab´)_2_. (DOCX 244 kb)

## Data Availability

All data generated or analyzed during this study are included in this published article [and its supplementary information files].

## Abbreviations

%ID/g: Percent injected dose/g
A_280_: Absorbance at 280 nm
Ã_s_: Time integrated activity in a source organ
ATCC: American Type Culture Collection
AUC: Area-under-the-curve
BGO: Bismuth germinate
B_max_: Maximum number of binding sites per cell
BOD: Biodistribution
CRT: Chemoradiotherapy
CT: Computed tomography
D: Mean dose
DOTA: 1,4,7,10-tetraazacyclododecane-1,4,7,10-tetraacetic acid
DOTA-NHS: DOTA N-hydroxysuccinimide ester
Eβ_max_: Maximum beta particle energy
Eβ_max_^+^: Maximum positron energy
EGFR: Epidermal growth factor receptors
F (ab’)_2_: Divalent antigen-binding fragment
Fab: Monovalent antigen-binding fragment
HER2: Human epidermal growth factor receptor-2
HNSCC: Head and neck squamous cell carcinoma
ID: Injected dose
IgG: Immunoglobulin G
IHC: Immunohistochemical
ITLC-SG: Instant thin layer silica gel chromatography
k: physical decay constant
K_d_: Dissociation constant
kDa: kiloDalton
keV: kiloelectron volt
mAb: monoclonal antibody
microPET/CT: micro positron emission tomography/computed tomography
microSPECT/CT: micro single photon emission computed tomography/computed tomography
MWCO: molecular weight cut-off
mSv: millisievert
NOD/scid: Non-obese diabetic/severe combined immunodeficiency
NOTA: 2,2′,2″-(1,4,7-triazacyclononane-1,4,7-triyl) triacetic acid
NRG: NOD-*Rag1*^*null*^ *IL2rg*^*null*^ (NRG)
NSB: Non-specific binding
OLINDA/EXM: Organ Level INternal Dose Assessment/ EXponential Modeling
p.i.: Post-injection
PBS: Phosphate-buffered saline
PDX: Patient-derived xenograft
PET: Positron emission tomography
RCP: Radiochemical purity
R_f_: Ratio to the solvent front
RICs: Radioimmunoconjugates
RIT: Radioimmunotherapy
ROI: Region-of-Interest
RT: Room temperature
S: Snyder factor
s: Seconds
s.c.: Subcutaneous
SB: Specific binding
SDS-PAGE: Sodium dodecyl sulfate-polyacrylamide gel electrophoresis
SE-HPLC: Size-exclusion high performance liquid chromatography
SEM: Standard error of the mean
SPECT: Single photon emission computed tomography
Sv: Sievert
T/B: Tumour/blood
TB: Total binding
UV: Ultraviolet
