# Supplementary material for: Dose predictions for [177Lu]Lu-DOTA-panitumumab F(ab′)2 in NRG mice with HNSCC patient-derived tumour xenografts based on [64Cu]Cu-DOTA-panitumumab F(ab′)2 – implications for a PET theranostic strategy
Source: EJNMMI Radiopharm Chem. 2021 Aug 12;6:25. doi: 10.1186/s41181-021-00140-1 (PMC8360260; doi:10.1186/s41181-021-00140-1)
Supplement: Supplementary file 1 — Fig. S1. SDS PAGE analysis of trastuzumab IgG and F(ab′)2 under non-reducing (lanes 1, 2, respectively) or reducing (lanes 3, 4, respectively) conditions on a 7.5% Tris/Glycine mini-gel stained with Coomassie blue. Table S1. Estimated radiation equivalent doses in a tumour-bearing NRG mouse for [64Cu]Cu-DOTA-panitumumab F(ab´)2. Table S2. Projected radiation equivalent doses for a female human adult with a 2 cm tumour in the neck for [64Cu]Cu-DOTA-panitumumab F(ab´)2 or [177Lu]Lu-DOTA-panitumumab F(ab´)2. (DOCX 244 kb) [file 41181_2021_140_MOESM1_ESM.docx]

**Dose predictions for [^177^Lu]Lu-DOTA-panitumumab F(ab′)_2_ in NRG mice with HNSCC patient-derived tumour xenografts based on [^64^Cu]Cu-DOTA-panitumumab F(ab′)_2_ – Implications for a PET theranostic strategy**

**Supplementary Information**

Anthony Ku ^1, †^, Misaki Kondo ^1, †^, Zhongli Cai ^1, †^, Jalna Meens ^2^, Min Rong Li ^1^, Laurie Ailles ^2,3^ and Raymond M. Reilly ^1,2,4,5^*

^1^ Department of Pharmaceutical Sciences, University of Toronto, Toronto, ON, Canada

^2^ Princess Margaret Cancer Centre, Toronto, ON, Canada

^3^ Department of Medical Biophysics, University of Toronto, Toronto, ON, Canada

^4^ Department of Medical Imaging, University of Toronto, Toronto, ON, Canada

^5^ Joint Department of Medical Imaging, University Health Network, Toronto, ON, Canada

^†^ Anthony Ku, Misaki Kondo and Zhongli Cai contributed equally to this work.

* Correspondence to: Raymond M Reilly; Email: [raymond.reilly@utoronto.ca](mailto:raymond.reilly@utoronto.ca)

Tel. 1-416-946-5522; FAX: 1-416-978-8511


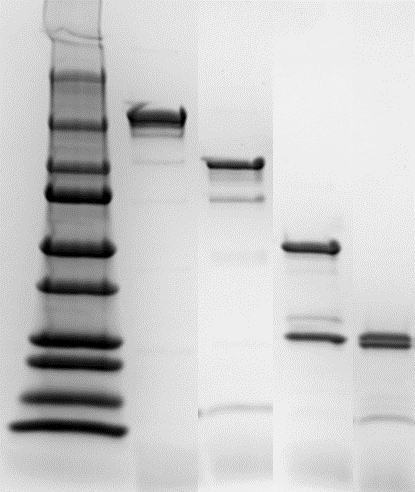


20

25

37

50

75

100

150

250

15

10

MW

1

2

3

4

Non-reducing

Reducing

kDa

**Fig. S1.** SDS PAGE analysis of trastuzumab IgG and F(ab′)_2_ under non-reducing (lanes 1, 2, respectively) or reducing (lanes 3, 4, respectively) conditions on a 7.5% Tris/Glycine mini-gel stained with Coomassie blue.

**Table S1**

Estimated radiation equivalent doses in a tumour-bearing NRG mouse for [^64^Cu]Cu-DOTA-panitumumab F(ab´)_2_.

|  | **Radiation equivalent dose^a^ (Sv/MBq)** | | |
| --- | --- | --- | --- |
| **Organ** | **^64^Cu/PET ^b^** | **^64^Cu/BOD ^c^** | **Comparison of doses for ^64^Cu estimated from PET or BOD**  **(*P*-value)** |
| Liver | 0.14 ± 0.02 | 0.10 ± 0.02 | n.s |
| Tumour ^d^ | 0.12 ± 0.04 | 0.14 ± 0.03 | n.s. |

^a^ The equivalent doses (D) were calculated as D = Ã_s_ × S × W_R_, where Ã_s_ is the time-integrated ^64^Cu activity in source organs and S are the Snyder values for ^64^Cu in mice (Bitar et al., 2007; Xie and Zaidi, 2013) and W_R_ is the radiation weighing factor.

^b^ The time-integrated radioactivity was calculated based on microPET/CT imaging studies of NRG mice injected i.v. (tail vein) with [^64^Cu]Cu-DOTA-panitumumab F(ab´)_2_.

^c^ The time-integrated radioactivity was calculated based on biodistribution (BOD) studies of NRG mice injected i.v. (tail vein) with [^64^Cu]Cu-DOTA-panitumumab F(ab´)_2_.

^d^ Estimated using the sphere model in OLINDA/EXM software based on the measured tumour mass (Stabin et al., 2005).

**Table S2**

Projected radiation equivalent doses for a female human adult with a 2 cm tumour in the neck for [^64^Cu]Cu-DOTA-panitumumab F(ab´)_2_ or [^177^Lu]Lu-DOTA-panitumumab F(ab´)_2_

|  | **Equivalent dose (mSv/MBq) ^a^** | | | | |
| --- | --- | --- | --- | --- | --- |
| **Target Organ** | **[^64^Cu]Cu-DOTA-panitumumab F(ab´)_2_** | | **[^177^Lu]Lu-DOTA-panitumumab F(ab´)_2_** | | **[^177^Lu]Lu-DOTA-panitumumab F(ab´)_2_** |
|  | BOD ^b^ | PET ^c^ | BOD ^b^ | PET ^c^ | BOD ^d^ |
| Heart | 0.031 ± 0.004 |  | 0.20 ± 0.04 |  | 0.19 ± 0.08 |
| Lung | 0.032 ± 0.003 |  | 0.28 ± 0.02 |  | 0.16 ± 0.02 |
| Liver | 0.065 ± 0.012 | 0.080 ± 0.009 | 0.70 ± 0.07 | 0.83 ± 0.10 | 1.05 ± 0.08 |
| Spleen | 0.030 ± 0.003 |  | 0.42 ± 0.12 |  | 0.39 ± 0.11 |
| Pancreas | 0.020 ± 0.003 |  | 0.14 ± 0.05 |  | 0.09 ± 0.02 |
| Stomach | 0.033 ± 0.002 |  | 0.18 ± 0.01 |  | 0.22 ± 0.02 |
| Intestines | 0.037 ± 0.002 |  | 0.26 ± 0.03 |  | 0.27 ± 0.03 |
| Kidneys | 0.043 ± 0.006 |  | 0.37 ± 0.05 |  | 0.44 ± 0.04 |
| Whole Body | 0.030 ± 0.002 |  | 0.15 ± 0.01 |  | 0.22 ± 0.01 |
| Tumour ^e^ | 0.092 ± 0.017 | 0.065 ± 0.016 | 1.10 ± 0.32 | 1.14 ± 0.22 | 1.47 ± 0.46 |

^a^ The equivalent doses were calculated using OLINDA/EXM software for a female human adult injected i.v. with [^64^Cu]Cu-DOTA-panitumumab F(ab´)_2_ or [^177^Lu]Lu-DOTA-panitumumab F(ab´)_2_. The time-integrated activity of ^64^Cu or ^177^Lu in source organs were projected from biodistribution studies or ROI analysis of microPET/CT images in female NRG mice bearing s.c. patient derived HNSCC tumours using the %kg/g method (Kirschner et al., 1973).

^b^ The time-integrated activity was projected based on the biodistribution (BOD) studies of NRG mice injected i.v. with [^64^Cu]Cu-DOTA-panitumumab F(ab´)_2_

^c^ The time-integrated activity was projected based on ROI analysis of the liver and tumour on microPET/CT studies of NRG mice injected i.v. with [^64^Cu]Cu-DOTA-panitumumab F(ab´)_2_.

^d^ The time-integrated radioactivity was projected based on the biodistribution (BOD) studies of NRG mice injected i.v. with [^177^Lu]Lu-DOTA-panitumumab F(ab´)_2_.

^e^ Assuming a 2 cm diameter spherical tumour in the neck.

**References**

Bitar, A., Lisbona, A., Thedrez, P., Sai Maurel, C., Le Forestier, D., Barbet, J., Bardies, M., 2007. A voxel-based mouse for internal dose calculations using Monte Carlo simulations (MCNP). Phys Med Biol 52, 1013-1025.

Kirschner, A., Ice, R., Beierwaltes, W., 1973. Radiation dosimetry of ^131^I-19-iodocholesterol. J. Nucl. Med. 14, 713-717.

Stabin, M.G., Sparks, R.B., Crowe, E., 2005. OLINDA/EXM: the second-generation personal computer software for internal dose assessment in nuclear medicine. J. Nucl. Med. 46, 1023-1027.

Xie, T.W., Zaidi, H., 2013. Assessment of S Values in Stylized and Voxel-Based Rat Models for Positron-Emitting Radionuclides. Mol. Imaging Biol. 15, 542-551.
